# Tone Language Speakers and Musicians Share Enhanced Perceptual and Cognitive Abilities for Musical Pitch: Evidence for Bidirectionality between the Domains of Language and Music

**DOI:** 10.1371/journal.pone.0060676

**Published:** 2013-04-02

**Authors:** Gavin M. Bidelman, Stefanie Hutka, Sylvain Moreno

**Affiliations:** 1 Institute for Intelligent Systems, University of Memphis, Memphis, Tennessee, United States of America; 2 School of Communication Sciences & Disorders, University of Memphis, Memphis, Tennessee, United States of America; 3 Department of Psychology, University of Toronto, Toronto, Ontario, Canada; 4 Rotman Research Institute, Baycrest Centre for Geriatric Care, Toronto, Ontario, Canada; University of Nevada, Las Vegas, United States of America

## Abstract

Psychophysiological evidence suggests that music and language are intimately coupled such that experience/training in one domain can influence processing required in the other domain. While the influence of music on language processing is now well-documented, evidence of language-to-music effects have yet to be firmly established. Here, using a cross-sectional design, we compared the performance of musicians to that of tone-language (Cantonese) speakers on tasks of auditory pitch acuity, music perception, and general cognitive ability (e.g., fluid intelligence, working memory). While musicians demonstrated superior performance on all auditory measures, comparable perceptual enhancements were observed for Cantonese participants, relative to English-speaking nonmusicians. These results provide evidence that tone-language background is associated with higher auditory perceptual performance for music listening. Musicians and Cantonese speakers also showed superior working memory capacity relative to nonmusician controls, suggesting that in addition to basic perceptual enhancements, tone-language background and music training might also be associated with enhanced general cognitive abilities. Our findings support the notion that tone language speakers and musically trained individuals have higher performance than English-speaking listeners for the perceptual-cognitive processing necessary for basic auditory as well as complex music perception. These results illustrate bidirectional influences between the domains of music and language.

## Introduction

A rapidly growing body of empirical evidence suggests that brain mechanisms governing music and language processing interact and might share an important link with respect to their underlying neurophysiological processing [1], [2], [3], [4], [5]. For example, neuroanatomical regions including Broca’s and Wernicke’s area and electrophysiological markers (N400 and P600) typically associated with language-specific operations (e.g., semantic/syntactic processing) are also recruited for processing the melodic and harmonic relationships of music [4], [5], [6]. In trained musicians, frontal regions (e.g., BA 47) typically associated with higher-order language comprehension, also show activation to the complex metric and rhythmic structures of music [7]. These studies provide evidence for a common neuronal mechanism subserving the temporal coherence found in both domains and demonstrate the intimate coupling between underlying neural processes recruited for language- and music-related processing.

Recognizing the shared brain structure between language and music leads to the provocative question of whether or not music ability might impact language-related abilities and vice versa. Indeed, the extensive overlap between these domains has led many to posit that musicianship and certain language backgrounds might impact processing in the complementary domain, i.e., so-called perceptual-cognitive “transfer effects” [3], [8], [9]. Such *cross-domain* influences have now been extensively reported in the direction from *music-to-language*. Musicians demonstrate perceptual enhancements in a myriad of language-specific abilities including phonological processing [10], verbal memory [11], [12] and verbal intelligence [13], formant and voice pitch discrimination [14], sensitivity to prosodic cues [15], detecting durational cues in speech [16], degraded speech perception [14], [17], second language proficiency [18], [19], and lexical tone identification [20], [21], [22]. These perceptual advantages are corroborated by electrophysiological evidence demonstrating that both cortical [23], [24], [25], [26], [27], [28] and even subcortical [3], [29], [30], [31] brain circuitry altered by long-term music training facilitates the sensory-perceptual and cognitive control of speech information. Musicians’ brain-behavior benefits for speech and language are, presumably, mediated by a series of enhancements to both sensory and cognitive mechanisms which operate at multiple tiers of the processing hierarchy that mediate a range of function from low-level auditory processing to higher-level aspects of cognition.

To account for such music-to-language effects, Patel [32] proposed a neurocognitive model to describe how speech processing benefits might arise due to the coordinated plasticity resulting from music training. According to the OPERA (Overlap, Precision, Emotion, Repetition, Attention) hypothesis, speech-related benefits in musicians are largely attributable to the extensive overlap in brain networks engaged during speech and music listening. As an auditory activity, music places higher demands on these shared networks than typical speech communication, allowing the pathways to function with a higher “precision” of processing. Assuming emotional engagement, sufficient repetition, and focused attention during learning, the neural plasticity engendered from music training acts to benefit speech processing by promoting an increase in the magnitude, resolution, and efficiency with which brain networks register and process salient acoustic information, music, speech, or otherwise. Although not explicitly developed in its inception, the OPERA framework makes no *a priori* assumption that music-language transfer should be exclusively unidirectional. Interestingly, the ingredients of the model (e.g., repetition, attention, increased sensory encoding precision) are also satisfied by forms of language expertise. Indeed, as with musical training, tone language experience (e.g., Mandarin Chinese [3], [33]) and bilingualism [34] have been shown to similarly affect the neural encoding and perception of behaviorally-relevant sound. These results, cast in the context of the OPERA framework, thus allow the possibility that cognitive transfer between music and language might be *bidirectional*, a point that has heretofore been largely untested ([9], p.340).

Despite its theoretical and practical significance, evidence for *language-to-music* transfer is scarce and conflicting [35], [36]. Most studies have focused on the putative connection between tone languages and absolute pitch (e.g., [37], [38]), a rare note naming ability irrelevant to most music perception/production ([39], p.26), or its effects on amusia [40], [41], another rare phenomenon which affects a listener’s processing, memory, and recognition for pitch. A handful of electrophysiological studies have demonstrated that relative to English-speaking controls, listeners fluent in Mandarin Chinese have improved sensory encoding of simple musical pitch patterns as evident by smoother, more robust pitch tracking in their scalp-recorded brainstem responses as well as their overall cortical response magnitudes [3], [33], [42]. In contrast, behavioral studies reveal contradictory effects, reporting either very weak [41], [42], [43] or no observable enhancement [33], [36], [44], [45] in these listeners’ nonlinguistic pitch perception abilities, music or otherwise. The failure of these behavioral studies to demonstrate a clear tone-language advantage in music perception might be due to a number of methodological issues including heterogeneity in the experimental group (e.g., pooling listeners across multiple language backgrounds [43]), the ecological validity of the “musical stimuli” [33], and/or differences in experimental tasks.

Given the inconsistencies of the extant literature, we aimed to test if listeners with tone-language expertise display similar performance to musically-trained individuals on measures of auditory pitch acuity, music perception, and general cognitive ability. We employ a cross-sectional design herein examining these “auditory experts” as it is a necessary first step to verify a bidirectional relationship between music and language prior to manipulating these variables (i.e., language experience/training) in a longitudinal study. In order to increase the possibility of identifying behavioral correlates of language-to-music influences, we aimed to recruit individuals with linguistic pitch expertise whose exposure to aspects of pitch would more closely approximate that gained via musical training. Cantonese serves as our point of departure given its intricate tone system. In contrast to Mandarin, the Cantonese tonal inventory consists of six contrastive tones, most of which are level pitch patterns minimally differentiable based on pitch height [46], [47]. Importantly, the proximity of tones is on the order of a semitone [48], i.e., 6% difference in frequency, which parallels the minimum distance between adjacent pitches found in music. Given their specialization in perceiving minute changes in steady-state, level pitch [46], [49], we reasoned that Cantonese listeners would show improvements in basic auditory (e.g., pitch discrimination) as well as music perception abilities relative to untrained listeners (English-speaking nonmusicians). Thus, we assess whether or not tone-language speakers show enhanced performance on measures of music perception. Furthermore, we compared the performance of Cantonese-speakers to that of musicians, in order to contrast the behavioral benefits engendered by these two distinct forms of auditory expertise. Both language and music training have also been implicated in improving executive processing [8]. Thus, in addition to assessing between-group perceptual differences, we also assessed performance on aspects of higher-order cognition, including general fluid intelligence and nonverbal working memory.

## Methods

### Ethics Statement

All participants gave their written, informed consent in compliance with an experimental protocol approved by the Baycrest Centre Research Ethics Committee. All participants were paid for their time.

### Participants

Fifty-four adults were recruited from the University of Toronto and Greater Toronto Area to participate in the experiment. All participants reported normal hearing sensitivity and no previous history of neurological or psychiatric illnesses. Each participant completed music [50] and language history [51] questionnaires to assess linguistic and musical background, respectively. English-speaking musicians (hereafter referred to as M) (*n* = 18; 13 female) were amateur instrumentalists with at least 10 years of continuous training in Western classical music on their principal instrument (*μ±σ*: 15.2±4.8 years), beginning at or before the age of 13 (7.4±2.8 years). All musician participants had formal private or group lessons within the past 5 years and currently played their instrument(s). These inclusion criteria are consistent with similar definitions for “musicians” used in many previous studies examining the neuroplastic effects of musical training [3], [14], [25], [30], [31], [52]. English-speaking nonmusicians (hereafter referred to as NM) (*n* = 18; 9 female) had no more than 3 years of formal music training on any combination of instruments throughout their lifetime (0.55±0.19 years) nor had received formal instruction within the past 5 years. Most English-speaking participants had some exposure to a *non-tone* (M: 83%; NM: 61%), second language (L2; mainly French and Polish) but were late learners and/or only mildly fluent in their L2. Cantonese-speaking participants (hereafter referred to as C) (*n* = 18; 11 female) were classified as late bilinguals, having not received formal instruction in English before the age of ∼7 (6.7±3.1 years) [3], [25]. They were born and raised in mainland China and reported using their native Cantonese on a regular basis (> 60%) throughout their daily activities. As with NM participants, Cantonese speakers had minimal musical training throughout their lifetime (1.1±1.5 years) and had not received formal instruction within the past 5 years. Importantly, NM and C did not differ in their minimal extent of music training [*F*(1,34) =  2.47, *p* = 0.13]. The three groups were closely matched in age (M: 22.9±4.5 years, C: 23.2±3.5 years, NM: 25.4±4.2 years; *F*(2,51) =  2.21, *p* = 0.12), years of formal education (M: 17.2±3.5 years, C: 16.1±2.2 years; NM: 17.6±2.7 years; *F*(2,51) =  1.42, *p* = 0.25), and were all strongly right-handed as measured by the Edinburgh Handedness inventory [F(2,51) =  0.071, *p* = 0.93] [53].

### Cognitive measures of working memory and general intelligence

#### Raven’s test

General fluid intelligence was measured with Raven’s Advanced Progressive Matrices [54]. Raven’s employs exclusively nonverbal material, and thus, is ideally suited for measuring an individual’s general cognitive ability without introducing potential confounds of cultural or social bias. Each trial consisted of a 3×3 matrix with line drawings depicting abstract patterns in all but the bottom-right cell. Participants were required to select the missing pattern from between 6 to 8 alternatives and were given 10 min to complete the 29-item battery. Items became progressively more difficult and required greater reasoning ability and intellectual capacity over the course of the test. Raw scores (number correct) were recorded and used in subsequent analyses.

#### Corsi Blocks

A digital implementation of the well-established Corsi blocks tapping test [55] was used to gauge each individual’s nonverbal short-term working memory (WM). On each trial, participants saw a 6×6 grid of grey squares on the computer screen. A memory sequence was then presented by briefly changing the color of certain boxes in various locations on the screen. Participants were required to recall the sequence with identical order by clicking on the target boxes. Sequence length gradually increased in set size from 2 to 8 items, becoming progressively harder over the time course of the task. Two repetitions were presented for each span length. The longest span-length correctly recalled was used to measure each individual’s visual (i.e., non-auditory) WM capacity.

### Pitch perception measures

#### F0 difference limens (F0 DLs)

**Figure 1:**
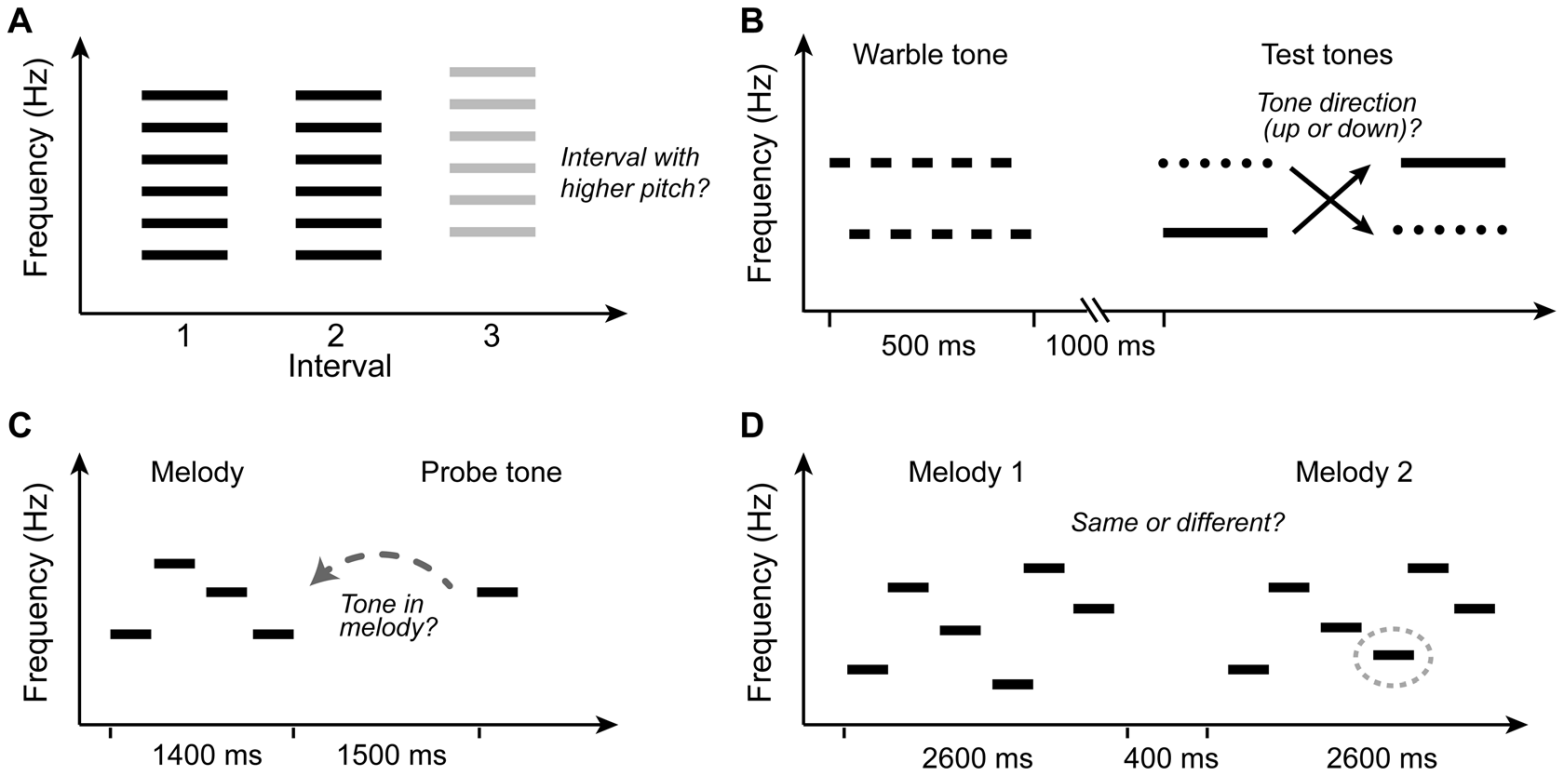
Schematic spectrograms of the various perceptual tasks used in the study . (A) Fundamental frequency difference limens (F0 DLs). Participants were instructed to detect the interval containing the higher pitch. (B) Pitch speed. Following a brief warble tone (distracter) and period of silence, listeners heard two pure-tones and were asked to identify their direction (i.e., ascending or descending). Test tone duration was varied adaptively to measure listener’s temporal threshold for identifying directional changes in pitch. (C) Pitch memory ability was assessed based on how well listeners judged whether or not a single probe tone had been heard in a preceding four-note melody. (D) Melody discrimination was measured by assessing how well listeners could discriminate short melodies that differed by as little as ½ or ¼ semitone (grey oval).

Behavioral fundamental frequency difference limens (F0 DLs) were measured for each participant using a three alternative forced choice (3AFC) discrimination task [14], [56]. For a given trial, participants heard three sequential intervals, two containing an identical reference complex tone (F0_ref_  =  220 Hz) and one containing a higher comparison, assigned randomly. The participants’ task was to identify the interval which contained the higher sounding pitch (Fig. 1A). Individual tones contained 10 harmonics of the fundamental, were 200 ms in duration, and separated by an interstimulus interval (ISI) of 400 ms. Discrimination thresholds were measured using a 2-down, 1-up adaptive paradigm which tracks 71% correct performance on the psychometric function [57]. The initial frequency difference between reference and comparison (ΔF0) was set at 20% of F0_ref_. Following two consecutive correct responses, ΔF0 was decreased for the subsequent trial, and increased following a single incorrect response. ΔF0 was varied using a geometric step size factor of two for the first four reversals and was decreased to √2 thereafter. Fourteen reversals were measured; the geometric mean of the last eight were used to compute each individual’s F0 DL for the run, calculated as the minimum percent change in F0 required to detect a change in pitch (i.e., ΔF0/F0_nom_). F0 DLs of three runs were averaged per listener to obtain a final estimate of each individual’s F0 discrimination threshold.

#### Pitch processing speed

We employed an auditory inspection time paradigm [58], [59] to measure listeners’ temporal threshold for resolving directional changes in pitch (hereafter referred to as “Pitch Speed”). On each trial, participants heard two pure-tones (784 Hz and 880 Hz) presented in random order and were asked to identify the direction of the pitch change (i.e., ascending or descending). Tone frequencies were selected such that they corresponded with musical notes of the Western scale (G5:784 Hz; A5 880 Hz). Each trial began with an initial warble tone consisting of the same two tones alternating rapidly (50-ms duration) for 500-ms, followed by 1s of silence, followed by the two test tones (Fig. 1B). Test tone durations were varied adaptively in a 3-down, 1-up tracking procedure (79% performance) to measure the shortest tone duration for which listeners could reliably identify the direction of pitch change. Following three consecutive correct responses, the duration of the two test tones was decreased for the subsequent trial; after one incorrect response, their duration was increased. Duration step size changed from a factor of 2 to √2 after the first four reversals. Nine reversals were measured, the last three of which were used to compute the listener’s threshold for the run. The average of three such runs was taken as the participants’ pitch speed threshold.

#### Pitch memory

We adopted a test of short-term pitch memory previously designed to test the relationship between musical and nonmusical cognitive abilities (cf. [60], [61]). The task assesses short-term memory of pitch sequences, and is a musical counterpart to the classic digit span test commonly employed to assess verbal working memory (e.g., [62]). On each trial, participants heard a short four-note melody (350-ms complex tones). Following a 1.5-s retention interval of silence, participants were asked to judge as quickly as possible whether or not a probe tone had been heard in the preceding sequence (Fig. 1C). Though four pitches is a rather short phrase to be considered a “melody”, pilot testing indicated even this few number of notes was sufficiently challenging for nonmusician listeners. Individual pitches were drawn randomly from the Western chromatic scale. Random selection ensured that melodies were tonally ambiguous thereby minimizing the chance that sequences could be recalled based on internalized labels (e.g., musical solfège: Do, Re, Mi, etc.). Participants heard 100 trials during the course of a run, half of which were catch trials, i.e., the probe tone did not occur in the melody. Behavioral sensitivity (*d'*) was computed using hit (*H*) and false alarm (*FA*) rates for each run (i.e., *d'*  =  *z(H)- z(FA)*, where *z(.)* represents the *z*-transform). Individual *d'* values were then averaged for two consecutive runs to obtain each subject’s overall pitch memory ability. Reaction time for correctly identified trials was also computed, measured as the time-lag between stimulus offset and the listener’s response.

#### Melody discrimination

A melody discrimination task was developed to probe pitch discrimination ability in musically-relevant contexts. For each run, participants heard forty pairs of short tonal melodies (6 notes each) separated by an ISI of 800 ms. Sequences were restricted in duration to minimize working memory effects on melody recognition [63]. Individual tones were 325 ms in duration and were composed of 10 harmonics of the F0. F0 frequencies were chosen to match pitches from a middle register of the diatonic musical scale [A4 (440 Hz) – A6 (1760 Hz)] and followed the prototypical voice-leading rules of Western music practice [64]. Half of the trials contained melody pairs that were identical; the other half contained melodies in which a single tone (random location) was detuned from what it was in its counterpart. Detuning was achieved by sharpening or flattening a single tone by ½ semitones (counterbalanced across “different” trials). Extensive pilot testing indicated this amount of detuning was sufficiently challenging for even musically trained listeners but avoided ceiling/floor effects. On each trial, participants were asked to indicate whether or not the melody pair was the same or different (Fig. 1D). The mean *d'* from three repetitions of the task were used to compute each listener’s melody discrimination sensitivity. To assess possible differential effects and behavioral limits between groups, participants also completed a version of the melody discrimination task in which the detuning was set at ¼ semitones. It should be noted that this minute deviation is well below any pitch differences typically observed in either Western music or between tones in the Cantonese tonal space [47], [48], [49].

Auditory stimuli were presented via circumaural headphones (Sony MDR V900HD) at a comfortable intensity (∼75 dB SPL). Individual tones contained 10-ms cos^2^ ramps to avoid spectral splatter and audible clicks in the stimuli. Stimulus presentation and response collection were implemented in custom GUI interfaces coded in MATLAB (The MathWorks). Feedback was provided for all pitch-related tasks. Task order of the battery was counterbalanced across participants according to a balanced Latin square design [65]. The experimental protocol took ∼2 hours to complete.

### Statistical analysis

For tasks measuring sensitivity (*d′*), cases where listeners obtain perfect accuracy (i.e., *H*  =  1, *FA*  =  0) implies a *d′* of infinity. In these instances, a correction was applied by adding 0.5 to both the number of hits and false alarms in order to compute a finite *d′* [66], [67]. With the exception of pitch memory, reaction time, Raven’s, and Corsi span, initial diagnostics revealed heteroscedasticity in the dependent variables. These values were consequently square root transformed to improve normality and homogeneity of variance assumptions necessary for parametric statistics. The choice of a square root transformation was based on output from the analytic Box-Cox procedure [68] which provides a formal, objective method to determine the power of the appropriate statistical transform [69]. Transformed dependent variables were then submitted separately to one-way, mixed-model ANOVAs. Group served as the fixed, between-subject factor (3 levels; M, C, NM) and participants as a random factor nested within group. An *a priori* level of significance was set at *α*  =  0.05. Multiple pairwise comparisons were adjusted with Tukey-Kramer corrections. Where appropriate, partial eta-squared (η^2^_partial_) values are reported to indicate effect sizes.

## Results

**Figure 2:**
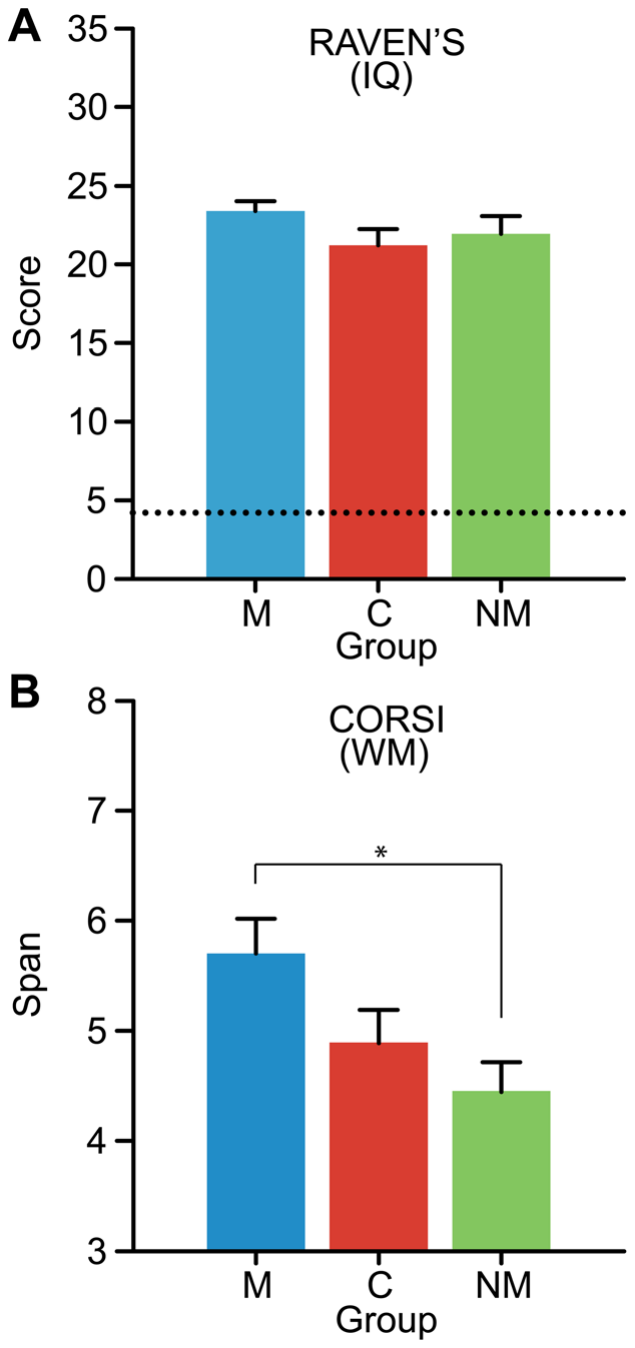
Group performance on measures of general cognitive ability . (A) Nonverbal fluid intelligence as measured by Raven’s Advanced Progressive Matrices. Dotted line denotes chance performance. (B) Spatial working memory span as measured by Corsi blocks. No group differences were observed for general intelligence but musicians showed better performance in working memory capacity as indicated by their larger memory span length relative to C and NM. Here and throughout, error bars  =  s.e.m., *p< 0.05, **p< 0.01, ***p< 0.001, M: Musicians, C: Cantonese, NM: English-speaking nonmusicians.

Cognitive measures of nonverbal intelligence and spatial working memory are shown in Fig. 2. No difference was observed between groups on Raven’s intelligence (*p* = 0.27). In contrast, an omnibus ANOVA revealed that Corsi WM span differed between groups [*F*(2,51) =  4.62, *p* = 0.014, η^2^_partial_ =  0.15]. Pairwise contrasts revealed this effect was largely attributable to larger WM capacity in musicians relative to nonmusicians (*p*<0.01) and a marginally significant effect between M and C (*p* = 0.06). The difference between C and NM listeners failed to reach significance.

**Figure 3:**
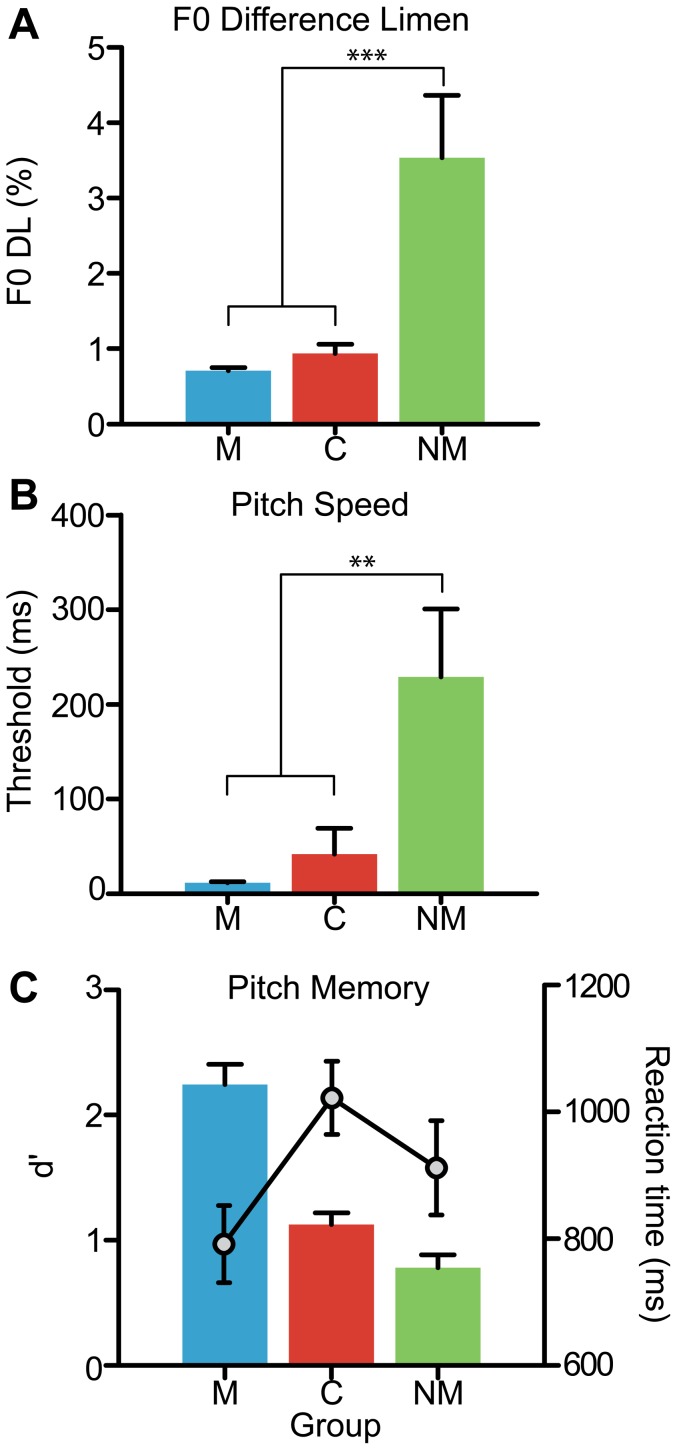
Group performance on basic psychoacoustic measures of auditory processing . (A) Fundamental frequency difference limens (F0 DLs) measure the smallest change in pitch listeners can reliably detect. (B) Pitch speed measures listeners’ temporal threshold for resolving directional changes in pitch. For both metrics, smaller values represent better performance. Pitch discrimination and speed of processing are markedly better in both musician and Cantonese-speaking participants indicating that both musical and linguistic pitch experience are associated with improvements in basic auditory acuity. No differences were observed between M and C. (C) Behavioral sensitivity (bars) and reaction time (lines) in recalling whether or not a single probe tone had occurred in the preceding tonally ambiguous melody. Relative to Cantonese-speaking listeners and nonmusician controls, musicians showed superior ability in both memory accuracy and speed of recall. Cantonese outperformed nonmusicians in accuracy but also suffered a time-accuracy tradeoff as indicated by their slower reaction times.

Group performance for psychophysical measures of basic auditory acuity is shown in Fig. 3. An ANOVA revealed a significant main effect of group on F0 DLs [*F*(2,51) =  14.57, *p*<0.001, η^2^_partial_ =  0.36] (Fig. 3A). Post-hoc multiple comparisons revealed that musician and Cantonese listeners obtained significantly better DLs than English-speaking nonmusicians (i.e., [M  = C] < NM; *p*<0.001), meaning they were better able to detect minute changes in pitch. C and M did not differ with respect to their pitch discrimination sensitivity (*p* = 0.59). On average, M and C DLs were ∼3.5 times smaller than those of NMs.

Similar results were found for pitch speed, which measures listeners’ temporal threshold for resolving directional changes in pitch (Fig. 3B). An omnibus ANOVA revealed a significant main effect of group on pitch speed [*F*(2,51) =  11.14, *p*<0.0001, η^2^_partial_ =  0.30] (Fig. 3B). As with F0 DLs, musicians and Cantonese listeners achieved better (i.e., smaller) discrimination thresholds than English-speaking NMs (*p*<0.01).

Short-term pitch memory, as measured by probe tone recall, is shown in Fig. 3C. A significant main effect of group was observed on both accuracy [*F*(2,51) =  40.65, *p*<0.0001, η^2^_partial_ =  0.61] as well as reaction time [*F*(2,51) =  3.44, *p* = 0.03, η^2^_partial_ =  0.12]. Post-hoc comparisons revealed a gradient in group performance (M > C> NM). Musicians were both more accurate and faster than C and NMs in identifying whether or not the probe had occurred in the preceding melody (*p*<0.001). Cantonese listeners also achieved better accuracy than nonmusicians (*p*<0.01) but at the expense of their reaction time, which was slower than that achieved by musicians (*p* = 0.03; i.e., time-accuracy tradeoff).

**Figure 4:**
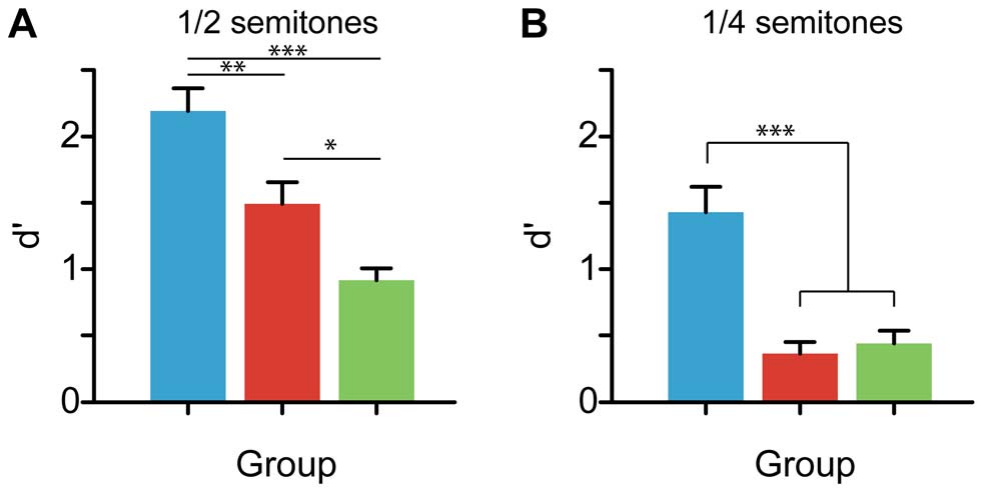
Group comparison of musical melody discrimination . (A) Sensitivity (*d′*) for discriminating melodies differing by ½ semitone. A systematic gradient is observed in performance across groups (M > C > NM). The fact that Cantonese participants outperform nonmusicians suggests that tone-language speakers have behavioral advantages in musical pitch perception. (B) Discrimination sensitivity for melodies differing by ¼ semitone. Musicians showed superior discrimination relative to Cantonese and nonmusicians; no group differences were found between C and NM in this difficult condition.

Performance for musical melody discrimination is shown in Fig. 4. Results of an ANOVA revealed a main effect of group for both the ½ semitone (Fig 4A; *F*(2,51) =  20.13, *p*<0.0001, η^2^_partial_ =  0.44) and ¼ semitone (Fig. 4B; *F*(2,51) =  17.81, *p*<0.0001, η^2^_partial_ =  0.41) conditions. Post-hoc contrasts again revealed a gradient in performance between groups for the ½ semitone condition (i.e., M > C > NM, all *p* <0.05), suggesting a perceptual advantage for musical pitch in both musically trained and Cantonese-speaking individuals relative to English-speaking nonmusicians. In contrast, while musicians continued to demonstrate robust discrimination performance in the challenging ¼ condition (M >> [C = M], *p*<0.001), the difficulty of the task greatly hindered performance for musically naïve participants (C and NM), who showed equally poor discrimination (*p* = 0.75). Together, these results suggest that relative to English-speaking nonmusicians, Cantonese speakers show improved perceptual abilities for aspects of musical pitch, but only when the demands of the task approximate the auditory experience of their native tone language—a ¼ semitone difference in pitch falls well below what would occur between successive tones in the Cantonese language [48].

**Figure 5:**
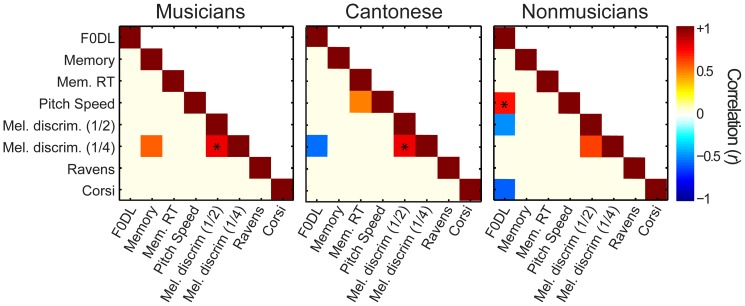
Correlations between perceptual and cognitive abilities . Cells of each matrix represent the correlation coefficient (Pearson’s *r*) between pairs of tasks where the color denotes the magnitude of correspondence. Warmer colors denote positive associations; cooler colors denote negative associations. Correlations are threshold at *p* < 0.05 (uncorrected) such that only significant cells are visible. Starred cells denote correlations surviving correction for multiple comparisons using a false discovery rate of *α*  =  0.05 [89].

To explore putative relationships between the degree of tone language or music training and cognitive/perceptual abilities, pairwise correlations (Pearson’s *r*) were conducted between all response variables (Fig. 5). As expected, correlations between performance on easy (½ semitone) and difficult (¼ semitone) melody discrimination conditions were apparent across the three groups. Yet, notable differences in correlation patterns were found between groups on other perceptual measures. For musicians, pitch memory positively predicted melody discrimination. Yet, only F0 DLs (i.e., basic pitch sensitivity) emerged as a reliable predictor of melody discrimination for C and NM groups. The association between pitch memory and melody discrimination for musicians but not musically naïve participants may reflect differences in listening strategy. For example, musicians might first assimilate sequences as a whole and then exploit auditory short-term memory to make judgments of melodies. In contrast, performance by nonmusicians (C and NM) might be limited by psychophysical constraints, such that their ability to hear out differences in melody is limited primarily by their ability to discriminate consecutive tones.

**Figure 6:**
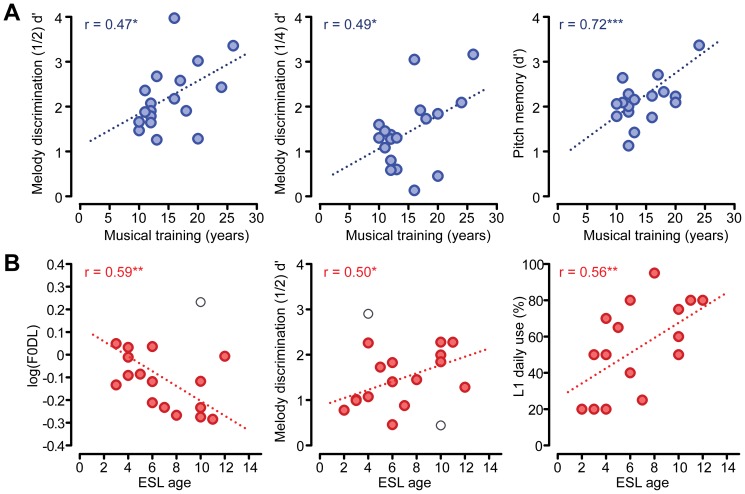
Scatter plots illustrating the correspondence between expertise and behavioral measures of musical pitch ability . (A) Musical training predicts easy and difficult melody discrimination performance (left and middle panels) as well as pitch memory ability (right panel). Positive associations indicate that recall and sensitivity for pitch patterns is sharpened with continued musical training. (B) English as a Second Language (ESL) age predicts Cantonese speakers’ pitch and melody discrimination performance (left and middle panels, respectively). ESL age is associated with the percentage of L1 daily use (right panel) such that late bilinguals (i.e., higher ESL age) continue to use their native Cantonese on a more regular basis than early onset bilinguals. As with musical training, extended experience with linguistic pitch appears to improve music perception ability. Open circles denote points deemed influential observations via Cook’s D [90] excluded from the regression analyses prior to least squares fitting. Stars denote uncorrected significance levels: *p< 0.05, **p< 0.01, ***p< 0.001. Note that given three tests per group (C, M), a Bonferroni corrected family-wise error rate of *α*  =  0.05 would require *p*< 0.0167.

Associations between task performance and musical/language expertise were also observed (Fig. 6). For musicians, years of musical training was a positive predictor of melody discrimination and pitch memory performance (Fig. 6A). No correlations were observed between years of musical training and Raven’s or Corsi scores. For Cantonese participants, English as a Second Language (ESL) was used as a proxy measure of tone language background and hence, linguistic pitch exposure; ESL corresponded well with first-language (L1) (i.e., Cantonese) daily use. As with musical training, ESL similarly showed positive associations with pitch perception abilities such that later onset of English—and hence longer exposure and use of the linguistic pitch patterns in Cantonese—was associated with superior perceptual performance (Fig. 6B). As with musicians, no correlations were detected between Cantonese participants’ ESL and the two non-auditory cognitive measures (i.e., Raven’s and Corsi). Collectively, these results highlight an association between a listener’s degree of tone language or music training and his/her perceptual abilities with music material.

## Discussion

By examining basic auditory as well as complex music perception in Cantonese, musician, and nonmusician listeners, we demonstrate that pitch expertise, whether originating from tone language or music, is associated with lower- (pitch discrimination sensitivity, processing speed) and higher-order (tonal memory, melodic discrimination) auditory processing necessary for robust music perception. Importantly, Cantonese participants outperformed their English-speaking nonmusician counterparts on nearly all measures of pitch and music perception, suggesting that tone language speakers have an advantage in processing the pitch information required for music listening. The strong correspondence between perceptual performance and a listener’s extent of speaking a tone language or musical training suggests that the benefits to music processing might be governed by the degree of plasticity acquired through long-term exposure to each of these auditory activities. Of theoretical and practical interest, the similarity in performance between Cantonese and musicians, together with the music-to-language effects demonstrated in previous studies (e.g., [3], [27], [70]), suggests that “transfer” between these domains is *bidirectional*. That is, a background in either domain might improve certain processing required by the other domain.

### Tone-language background and musicianship predict auditory perceptual abilities

Interestingly, we find significant correspondence between both the length of exposure to music or a tonal language and behavioral measures of musical pitch ability. For musicians, years of musical training was positively associated with melody discrimination as well as pitch memory performance (Fig. 6A) implying that duration of continued music training can predict a listener’s recall and sensitivity for behaviorally relevant pitch patterns. Similarly, ESL onset age was positively associated with Cantonese speakers’ pitch and melody discrimination abilities (Fig. 6B) suggesting that the more exposure to the lexical tones of the Cantonese language (i.e., later ESL onset), the better one’s acuity for musically-relevant sound.

Limitations of these findings are worth mentioning especially with respect to the correlational nature of our results. Every attempt was made to control latent subject factors (e.g., age, education, intelligence) within and between groups while allowing only the form of pitch expertise to vary between cohorts. Unfortunately, our study does not provide definitive conclusions as to whether the observed benefits in Cantonese and musicians results from linguistic/musical experience *per se*, or other, preexisting differences between groups. A cross-sectional design is a necessary first step to investigate the existence of bidirectionality between music and language but longitudinal studies are needed to determine if these findings truly stem from experience/training. It is possible, for example, that innate cultural or genetic differences might also contribute to the group differences we observe in perceptual-cognitive abilities. Indeed, recent work demonstrates a link between two genetic markers and both the incidence [71] and perception [72] of lexical tones in certain populations. Thus, Cantonese speakers may possess perceptual advantages in pitch processing irrespective of their specific language experience. Similar arguments could be made for the musicians in the present study [73].

Unfortunately, only randomized, longitudinal training studies can tease apart such “nature” and “nurture” contributions. Emerging evidence using such rigorous experimental manipulations have revealed differences in brain physiology and morphology subserving auditory processing following even relatively short-term training (< 1 month [13]; 15 months [74]) in children randomly assigned to music lessons relative to controls. Importantly, these effects also remain intact even after controlling for potential confounding factors [e.g., age, socioeconomic status (SES)] thereby isolating training as the key ingredient mediating the observed auditory plasticity [74], [75]. Similar benefits have also been demonstrated in the language domain following intense training in a tone language [76]. SES was not explicitly measured in the present study. However, participants were closely matched in their overall level of education and our data showed no significant difference in fluid intelligence score between our groups, as measured by Raven’s Matrices (see Fig. 2). Insomuch as it is possible, these controls help to minimize the chance that differences in perceptual abilities we observe between groups are the consequence of latent, preexisting factors. If cultural, genetic, or social factors do account for the present results, then whether advantage in music-related processing is caused by *experience* with a tone-language system would remain to be investigated. Regardless of the underlying mechanism, the findings remain that our cohort of tone language speakers and musicians show higher performance in general musical pitch processing and some aspects of higher-order auditory skills (e.g., WM).

### The hierarchical nature of music-language bidirectionality: Perceptual and cognitive abilities

Relative to nonmusicians, musicians and Cantonese participants demonstrated basic perceptual advantages, obtaining smaller (i.e., better) fundamental frequency difference limens and pitch-speed thresholds (Fig. 3A–B). These results suggest greater sensitivity and efficiency of basic auditory processing in tone language and musicians relative nonmusicians. In addition to these effects, we also found evidence for superior higher-level cognitive abilities in the Cantonese and musically-trained participants, as compared to nonmusicians. Despite controlling for group differences in age, education, and general IQ, superior performance was observed in improved tonal working memory (WM) in musicians and Cantonese relative to English-speaking nonmusicians (Fig. 3C). These results extend recent reports documenting enhanced auditory WM in musicians [77], [78], [79] by demonstrating similar effects in tone language speakers.

Interestingly, we also found that musicians demonstrated advantages in non-auditory WM (as measured by Corsi span) compared to Cantonese and nonmusician participants. Recent reports have been indecisive regarding the effects of musicianship on visual aspects of WM (cf. [77], [79]). The positive effect observed in the present study in contrast to previous reports may be due to differences in subject demographics. Indeed, all of the musicians in the current study were instrumentalists, the vast majority of whom were either pianists or violinists (55.5% and 44.4% of the sample, respectively). These instruments require the continuous mapping and recall of spatial location along the keyboard/fretboard to execute the correct order of notes. Thus, the association between musicianship and Corsi visuospatial WM we find may be due to the fact that most of our musicians commonly operate with abstract visual rules in their music practice and these abilities are subsequently revealed in the Corsi test. In contrast, despite their superior *auditory* WM (Fig. 3C), only a trend for *visual* WM advantage was observed for Cantonese speakers relative to nonmusicians—this effect was not significant. In addition, we failed to find a correspondence between ESL onset age (or years of musical training) and visual WM Corsi scores. This finding may indicate that the differences between groups in WM are independent of auditory expertise, *per se*. While the present correlational data do not allow us to definitively rule out preexisting differences in WM capacity between groups, it is also possible that the tone language expertise may not enhance aspects of non-auditory WM to the same degree as musical training. Future work is needed to clarify the potential effects of musicianship and tone language background on WM abilities.

Nevertheless, our results indicate that in addition to simple perceptual benefits, both musicians and tone language speakers show advantages in auditory processing requiring high-order operations (e.g., auditory WM). These behavioral results are supported by recent neuroimaging studies which reveal enhanced function in global brain networks subserving cognitive control, WM, and executive processes in trained musicians and bilinguals, as compared to monolingual nonmusicians [13], [80], [81]. Collectively, our findings suggest that auditory proficiency in the form of tone language or musical training yields enhancements to multiple levels of processing, impacting both sensory-perceptual [14], [29], [30], [31], [82], [83] and higher-order cognitive mechanisms [8], [13], [74], [84].

### Bidirectional music-language “transfer effects” are differentially weighted

To date, inconsistencies among previous studies have painted a clouded picture on the existence of language-to-music “transfer effects” [33], [41], [43]. Our results offer new insight into the nature of such effects by demonstrating a clear advantage in music-related processing in native tone language speakers. Importantly, Cantonese participants in our sample lacked any formal musical training. Yet, relative to controls, they showed superior performance on aspects of music perception including simple pitch discrimination, tonal memory, and melody perception. These effects also cannot simply be attributable to differences in “bilingualism” *per se,* as the vast majority of our subject pool, including English-speaking nonmusician controls, also had experience with a second (though non-tonal) language. Thus, we infer that the perceptual superiority observed in the Cantonese (and musical) group are the consequence of these specific forms of pitch expertise and not, for instance, the result of a latent “bilingualism factor”. The positive benefits for musical processing observed in our Cantonese group speaks to the potential for specific linguistic pitch abilities to carry over into nonlinguistic (i.e., musical) domains (cf. [3], [33], [41]). Integrated with the already well-documented influence of music on language processing [14], [27], [29], [85], [86], our results offer empirical support for the reverse effect and demonstrate the *bidirectional* nature between these two important domains of human cognition (cf. [32]).

A direct comparison between Cantonese and musicians’ performance helps further clarify this bidirectionality. Thresholds for pitch discrimination (F0 DLs) and processing speed were equally good among Cantonese and musician listeners relative to English-speaking nonmusicians (Fig. 3A-B), suggesting enhancements in basic pitch discrimination in these groups. These behavioral findings are supported by recent cross-domain ERP studies from our lab and others, which demonstrate that both tone language speakers and musicians have superior sensory encoding of pitch-relevant information at both subcortical [3], [33] and cortical [25], [27] levels of auditory processing. Yet, specific listening benefits for each group begin to deviate when considering more complex aspects of pitch and music perception. Indeed, despite their superior ability compared to controls, musicians outperformed Cantonese speakers on three of the five auditory tasks. These gradations in group performance (M > C > NM) were observed in time-accuracy measures of pitch memory (Fig. 3C) and musical melody perception (Fig. 4). Such tasks are arguably more demanding, requiring perceptual as well as cognitive resources (e.g., working memory; [63], [87]) to execute successfully. Thus, while Cantonese and musicians might share similar benefits in low-level pitch perception, it appears that tasks requiring higher-level contextual processing reveal that these gains could be hierarchical (cf. [33]). Restated within a framework of bidirectional “transfer” (i.e., [32]), the influence of one domain on another might be differentially weighted depending on the complexity of processing, the context and functional relevance of the auditory signal in question, and the degree of the listener’s specific expertise.

Of interest for future studies would be the inclusion of a tone language musician group, which would allow for the assessment of potential synergistic effects of linguistic and musical pitch expertise on auditory abilities. For example, Cooper & Wang [52] have recently reported on the combined effects of tone language and musical training on non-native lexical tone recognition in a word learning task. While this study examined aspects of language rather than music perception, similar conclusions were drawn as both musicians and tone language speakers demonstrated enhancements to linguistic pitch perception. Yet, no combined effect of music and tone language background was found. That is, the mixture of the two experiences did not provide an additional behavioral advantage above and beyond what either experience provided in and of itself [52]. In some sense, a ceiling effect might be reached with one intense auditory expertise such that adding the other does not produce any additional benefit. As such, it is unlikely that the inclusion of a Cantonese musician group in the current framework would have altered our results or conclusions. Indeed, such a group may have only acted to cloud interpretation, as it would be unclear whether the behavioral enhancements were attributable to the language or rather music history. Given this aliasing of experiential factors, as in previous studies [3], [33], [43], we purposely avoided including a group of Cantonese musicians to prevent confounding our interpretations. Nevertheless, future studies are needed to disentangle how these two important forms of experience/training contribute to auditory perceptual abilities [52], as well as further characterizing the role of experience and cultural influence on sensory and cognitive functions [41].

### Overlap is necessary but not sufficient for a music-language bidirectional relationship

The observed transfer in previous studies and our bidirectional evidence further corroborates a shared resources hypothesis, suggesting that the domains of language and music, at least at a certain level, recruit similar and overlapping functional brain networks [1], [32]. While neuroanatomical overlap is presumably a necessary prerequisite for cognitive transfer between activities, our data indicate it is not an entirely sufficient ingredient in and of itself. Rather, current findings suggest that in addition to overlap, functional enhancement (i.e., transfer) is most apparent when a listener’s experience is compatible with the acoustic demands of the domain in question [33]. This is most evident in the pattern of performance for tone language listeners and the gradient in performance observed across groups (e.g., M > C> NM). Relative to nonmusicians, Cantonese participants did show heighted music discrimination but only for relatively large pitch incongruency (Fig 4A); performance benefits were not observed for more subtle deviations, as Cantonese participants failed to detect ¼ semitone mistunings (Fig. 4B). Typically, pitch differences between tones in Cantonese are typically on the order of 0.5–1 semitones [47], [48], [49]. The absence of a perceptual benefit in the latter condition may reflect these listeners’ naivety to such minute changes in pitch which bear no behavioral relevance in their native Cantonese tongue. A similar argument might explain the inconsistency of previous behavioral studies to observe a music listening benefit in speakers of Mandarin Chinese. Because the Mandarin linguistic system is comprised nearly exclusively of curvilinear lexical tones, the discrete, level pitch patterns of music are similarly incompatible with these listeners’ experience and thus, no music benefit is observed [33], [43]. In some sense, the auditory demands required by music may simply fall outside the “scope” of some types of tone language experience. Alternatively, despite some shared cognitive and neural substrates, the way pitch information is handled in music may differ enough to require parallel but complementary mechanisms not tapped under language [88]. In other words, the two domains may not be entirely isomorphic. Thus, musicians’ superior ability to detect minute incongruences (Fig. 4B) may reflect the higher demands music places on fine pitch processing networks which is necessary, among other things, for detecting deviations in melody intonation [83].

## Conclusion

Taken together, our findings lead us to re-conceptualize the relationship between music and language as more than binary operations along common, shared brain pathways. Rather, we view these cross-domain influences as a continuum where the degree of “transfer” depends on how commensurate the acoustic demands of the listening task in question are with the cues found in the listener’s domain of expertise (i.e., their native language or music practice). More broadly, our findings offer new paths for conceptualizing learning and rehabilitation programs aimed at improving specific perceptual-cognitive function. Indeed, the bidirectionality observed in the present and previous work suggests that benefits targeted in one domain (e.g., increased music appreciation for cochlear implant users) might be garnered via carefully designed training regimens in a complementary domain (e.g., speech listening tasks), and vice versa.
